# From aMCI to AD: The Role of Visuo-Spatial Memory Span and Executive Functions in Egocentric and Allocentric Spatial Impairments

**DOI:** 10.3390/brainsci11111536

**Published:** 2021-11-19

**Authors:** Tina Iachini, Francesco Ruotolo, Alessandro Iavarone, Michele Carpinelli Mazzi, Gennaro Ruggiero

**Affiliations:** 1 Department of Psychology, Università degli Studi della Campania “L. Vanvitelli”, 81100 Caserta, Italy; francesco.ruotolo@unicampania.it (F.R.); gennaro.ruggiero@unicampania.it (G.R.); 2 Laboratory of Clinical Neuropsychology, Neurological Unit of “Ospedali dei Colli”, 80131 Naples, Italy; aleiavarone@gmail.com (A.I.); michelemm@hotmail.it (M.C.M.)

**Keywords:** visuo-spatial abilities, frontal functions, attentional resources, egocentric/allocentric frames of reference, healthy aging, amnesic mild cognitive impairment, Alzheimer’s disease

## Abstract

A difficulty in encoding spatial information in an egocentric (i.e., body-to-object) and especially allocentric (i.e., object-to-object) manner, and impairments in executive function (EF) are typical in amnestic mild cognitive impairment (aMCI) and Alzheimer’s disease (AD). Since executive functions are involved in spatial encodings, it is important to understand the extent of their reciprocal or selective impairment. To this end, AD patients, aMCI and healthy elderly people had to provide egocentric (What object was closest to you?) and allocentric (What object was closest to object X?) judgments about memorized objects. Participants’ frontal functions, attentional resources and visual-spatial memory were assessed with the Frontal Assessment Battery (FAB), the Trail Making Test (TMT) and the Corsi Block Tapping Test (forward/backward). Results showed that ADs performed worse than all others in all tasks but did not differ from aMCIs in allocentric judgments and Corsi forward. Regression analyses showed, although to different degrees in the three groups, a link between attentional resources, visuo-spatial memory and egocentric performance, and between frontal resources and allocentric performance. Therefore, visuo-spatial memory, especially when it involves allocentric frames and requires demanding active processing, should be carefully assessed to reveal early signs of conversion from aMCI to AD.

## 1. Introduction

Amnestic mild cognitive impairment (aMCI) is a clinical condition characterized by alterations in memory domains and a high risk of neurodegenerative progression [1,2]. Epidemiological studies have revealed that people with aMCI progress to Alzheimer’s disease (AD) dementia at a 4–10 times higher risk than healthy elderly people [1,3,4]. Conversion from aMCI to AD implies impairments in cognitive domains that may appear within a decade, with deficits in episodic and semantic memory, executive function, visuo-spatial memory, spatial skills, attention, apraxia, perceptual speed and verbal recall, up to the involvement of all cognitive domains in overt AD [5,6,7]. 

In the attempt to identify prodromal cognitive signs of AD onset, separate clinical observations have reported impairments of the executive function (EF) in aMCI and AD (for MCI: [8,9], for AD: [10,11,12]; for a review, see [13]). For example, Traykov et al. [14] found that task-switching and response inhibition abilities were decreased in MCI patients (see also [15]). Zhang et al. [16] reported that the aMCI group had difficulties compared with the healthy control group on tests of cognitive planning (e.g., Trail Making, verbal fluency tests) but not inhibition and control (Go/NoGo and Stroop tests). Gu et al. [9] observed more severe alterations in updating operations of working memory (WM), detections of the target stimulus and conflict processes in multiple domain-aMCI, compared to single-domain-aMCI patients and healthy controls. In stark contrast, Bisiacchi et al. [17] showed preserved EF in aMCI patients compared to healthy elderly individuals.

We argue that the reason for these conflicting results across samples of aMCI may be due to the complexity of EF and the concomitant involvement of visuo-spatial deficits. 

EF is a multi-componential cognitive ability consisting of cooperating processes that are necessary to acquire, combine and select spatial information and related processing strategies, and to plan and monitor behavioral motor responses according to environmental requirements [18,19]. These abilities are strongly implicated in processing egocentric (subject-to-object) and allocentric (object-to-object) visuo-spatial memory representations [20,21,22,23,24]. Indeed, a largely neglected aspect of the clinical assessment is that executive functions may be involved at various levels in egocentric and allocentric spatial encodings, and thus it would be important to understand the extent of their reciprocal or selective impairment [8,25,26].

Several studies have shown that deficits in egocentric and allocentric spatial representations, with a prevalence of the allocentric component, characterize the early stages of AD dementia and clinical phases (MCI) [2,27,28,29,30,31,32].

Research has documented strong associations between egocentric encoding and executive functions [18,23,24,33,34,35,36]. In addition, research has reported allocentric, and even egocentric, visuo-spatial memory deficits in aMCI that may precede the episodic and semantic memory difficulties usually observed in AD by as much as ten years [21,28,29,37,38,39,40,41].

Therefore, separate lines of research show a relationship between alterations of egocentric and allocentric spatial memory capacity, on the one hand [18,39,42] and executive functions, on the other hand. Previous evidence has indeed found that working memory impairments might be particularly sensitive for aMCI diagnosis [8,43,44] and that visuo-spatial working memory (VSWM) is more susceptible to AD-like neuropathology with respect to visual working memory (VWM) [45].

Building on this, here we aimed to understand the role of executive functions in the egocentric and allocentric spatial performance of AD patients, aMCI and healthy elderly people. Therefore, we examined three main subcomponents that could be closely linked to the typical functions of the egocentric and allocentric encodings: visual attention and planning (The Trail Making Test, TMT [46]), visuo-spatial working memory for temporary maintenance and manipulation of spatial information (Corsi Block Tapping Test Forward and Backward), and the inhibition of prepotent responses and monitoring (Frontal Assessment Battery, FAB; [47]). 

Regarding the spatial performance, we used a simplified computerized version of the Ego-Allo task that required egocentric/allocentric verbal judgments of relative distances between memorized stimuli [20,48,49]. 

We expected a worse performance of AD patients in egocentric and allocentric judgments. On the basis of previous literature, we expected that the allocentric performance should be significantly worse in AD patients than healthy people, but not aMCI people. Moreover, multiple regression analyses should clarify the role of attentional-executive and visuo-spatial resources in egocentric and allocentric performance.

## 2. Materials and Methods

### 2.1. Participants

The sample size was calculated with G*Power on the basis of the effect sizes observed in a previous study in which the same categories of participants and similar tasks were used [21]. Results showed that thirty-three participants in total were sufficient to detect an effect size (Cohen’s f) = 0.57. Eventually, the sample size included fifty-two participants as follows: eleven early Alzheimer’s disease patients (AD) (7 males; age range: 56–80, M = 69.7, SD = 7.56; education years M = 10.72, SD = 4.73) and ten aMCI patients (7 males; age range 61–79, M = 73.6, SD = 6.07; education years M = 8.5, SD = 2.59) were recruited at Ospedale dei Colli Aminei, C.T.O (Napoli, Italy). Thirty-one healthy elderly people (Control group, NC; 14 males; age range: 62–82, M = 70.71, SD = 5.08; education years M = 11.93, SD = 4.39) were recruited in seniors’ centers in the city of Naples (Italy). The NC group matched aMCI and eAD patients in terms of age and education. All participants voluntarily took part in the experiment and provided their informed consent. All participants were right-handed and had normal or corrected-to-normal vision. Recruitment and testing were in conformity with the local Ethics Committee requirements and the 2013 Helsinki Declaration.

Regarding the aMCI group, participants met the criteria for diagnosis of aMCI on the basis of the MCI working group of the European Consortium on AD [50]. The Mini-Mental State Examination (MMSE [51,52]) mean score was 25.9, SD = 2.13 (corrected score was 26.89, SD = 2.02). Patients with multiple domains or single non-memory domain MCI were not enrolled. In regards to the mild probable AD group, patients met criteria of NINCDS-ADRDA for AD [53]. The mean MMSE score was 20.27, SD = 3.46 (corrected score was 20.67, SD = 3.32). NC group average score at MMSE was 28.9, SD = 2.03 (corrected score was 27.59, SD = 1.67). The three groups did not differ significantly in either age (F= 1.28, *p* = 0.29) or years of education (F = 1.91, *p* = 0.16). As expected, the three groups differed significantly on the MMSE corrected score (F (2, 49) = 42.09, *p* < 0.001): the AD group performed worse than both aMCI and NC groups (at least *p* < 0.0001); whereas no difference between aMCI patients and NC group appeared (*p* = 0.65).

### 2.2. Experimental Sessions

The study comprised two sessions. In the first session, participants were administered three neuropsychological tests, the Frontal Assessment Battery (FAB), Trail Making Test (TMT) and Corsi Block Tapping Test (CORSI). In the second session, participants’ ability to represent spatial information according to an egocentric or allocentric reference system was assessed using the Ego-Allo Task [48].

#### 2.2.1. Session 1. Neuropsychological Assessment

FAB. The Frontal Assessment Battery (FAB), Trail Making Test (TMT) and Corsi Block Tapping Test were administered to all participants. The testing was carried out in a soundproofed, comfortable room.

FAB. The Frontal Assessment Battery (FAB) [47,54] is a short cognitive and behavioral test used to evaluate executive functions. Specifically, the FAB consists of six subtests that explore conceptualization (similarity), mental flexibility (phonemic fluency), motor programming, sensitivity to interference (conflicting instructions), inhibitory control (go-no-go) and environmental autonomy (prehension).

TMT. The Trail Making Test (TMT) [46] is a neuropsychological test used to assess visual attention (part A) and task switching (part B); it is also sensitive to detect cognitive impairment associated with dementia. In this experiment, we administered only part A, which evaluates, in particular, the visual-spatial detection capability, numeric recognition, visual-motor coordination and tracking speed. Participants had to link the numbers from 1 to 25 as fast as possible. The scoring is the number of seconds the participants take to complete the task. 

Corsi Block Tapping Test. This test assesses the visual-spatial working memory span [55]. The test consists of a wooden board containing nine blocks numbered 1 to 9 on the side of the experimenter. The experimenter taps a sequence of blocks, and the subjects are instructed to tap the same blocks immediately afterward. The sequence starts out with two blocks and then becomes more complex. Participants have to successfully reproduce at least two trials out of three per sequence length; otherwise, the administration stops and the span capacity is determined. In this study, we assessed both the forward and backward versions of block tapping. In the forward condition, the participant had to tap the same blocks that the experimenter had tapped earlier in the same sequence. In the backward condition, the participant had to tap the blocks backward from the last one to the first one.

#### 2.2.2. Session 2. Ego-Allo Task

The Ego-Allo task measures people’s ability to represent spatial information using an egocentric or an allocentric frame of reference. Specifically, participants are required to memorize the position of geometric objects placed in front of them and, once the objects are removed, they have to provide egocentric (e.g., “Which was the object closest to you?”) or allocentric (e.g., “Which was the object closest to object X?”) judgments [48]. For the current study, we used a simplified version of the Ego-Allo task (see below). 

### 2.3. Setting and Materials

The experiment was carried out in a soundproofed, comfortable room. The Ego-Allo task was built and administered using 3-D Vizard Virtual Reality Software Toolkit 5 (Worldviz, LLC, Santa Barbara, CA, USA). Participants were seated in a chair approximately 50 cm from a 15.6’ desktop monitor where stimuli were presented. 

**Figure 1:**
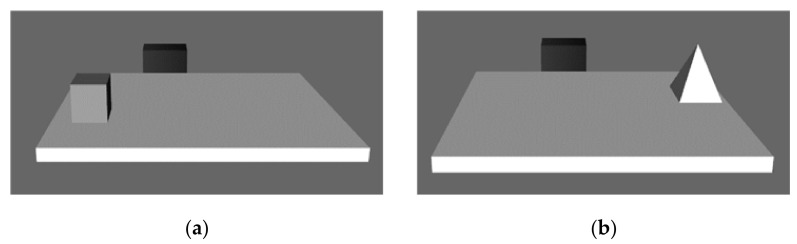
The figure shows an example of the two pictures that appeared in a trial, one after the other. (**a**) There is a cube closer to the participant than to the black bar. (**b**) There is a pyramid closer to the black bar than to the participant.

Stimuli were characterized by images of 3D geometrical objects (i.e., cube, pyramid, sphere and cone) placed on a table. The table presented a black bar on the side opposite to the viewer (see Figure 1). This black bar constituted the allocentric point of reference. The geometrical objects varied in color (dark/medium/light gray) and size: big objects (8 × 8 cm) and small objects (6 × 6 cm). The images were created with the 3D modeling software Sketchup Pro 2018 based on those used by Iachini and colleagues in several previous studies [21,37,38,48,49,56,57,58,59]. 

Participants were shown a tabletop, two geometric objects and a black bar (see Figure 1). For each trial, participants saw two pictures in sequence. For example: if in the first image the geometric object (e.g., the cube) was located 20 cm from the black bar, in the second image, the object (e.g., the pyramid) could be located 10 cm from the black bar. In this way, one object was closer to the participant, and the other object was closer to the black bar (see Figure 1).

**Figure 2:**
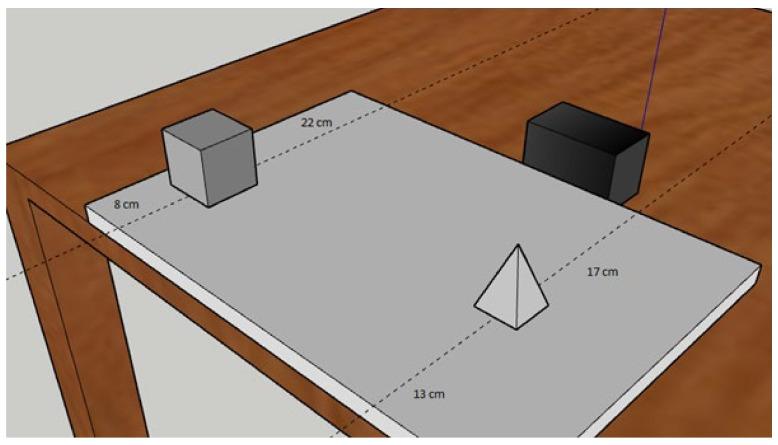
This image is only intended to show how the difficulties were calculated within each trial. As can be seen, the metric difficulty with respect to the participant’s position (shown in the image by the edge of the table) was 5 cm (the cube was positioned at 8 cm from the participant and the pyramid at 13 cm), the same level of metric difficulty results with respect to the black bar (22−17 = 5 cm).

More importantly, the two geometric objects were positioned in such a way as to achieve three levels of metric difficulty. The metric difficulty resulted from the difference between the position of the object and the frames of reference. For example, if one object was at 8 cm and the second object was at 13 cm from the participant, the metric difficulty for the egocentric judgment was 5 cm (13−8 = 5). For the same trial, the allocentric judgment had the same difficulty: one object was at 22 cm, and the second object was at 17 cm from the black bar (22−17 = 5 cm) (see Figure 2 for an example). Three levels of metric difficulty were used: easy = 11 cm; medium = 8 cm; difficult = 5 cm. Each trial had its specific metric difficulty (i.e., could be easy, medium or difficult).

A total of 72 trials were conducted; 36 trials were characterized by pairs with cube and pyramid, while the other 36 trials were characterized by pairs with sphere and cone. Considering the 36 trials with cube and pyramid, 18 trials were used in the egocentric condition (6 trials for each level of metric difficulty) and the other 18 in the allocentric condition (6 trials for each level of metric difficulty). The same applied to the trials with sphere and cone. 

### 2.4. Procedure

Participants were provided with written instructions that were then revised orally by the experimenter. 

Before beginning the Ego-Allo task, participants were shown the geometric objects one at a time and asked to name them. Once the objects had been accurately named, the training phase started (6 trials in total). During the training, participants were instructed on how to use each key of a keyboard to answer the questions; only the keys useful for the task were visible. Once the training phase was completed, participants started the testing phase.

**Figure 3:**
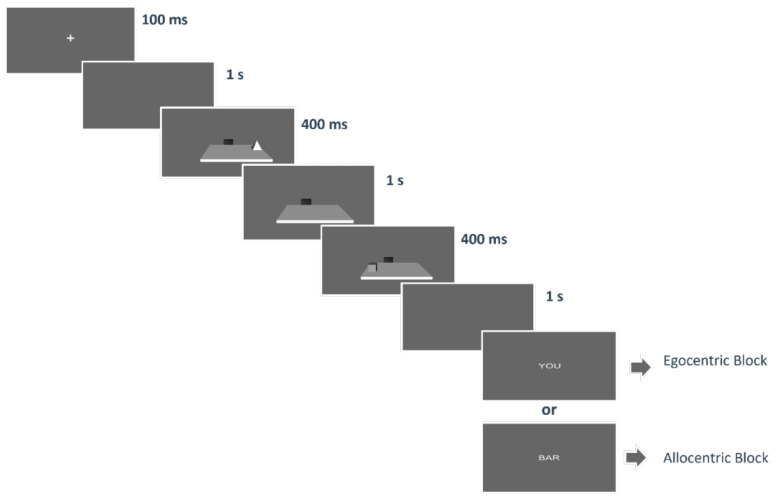
The figure shows a schematic representation of the sequence of events of a trial.

Each trial started with the presentation of a fixation cross on a grey screen for 100 ms; immediately after, a blank screen was presented for 1 s; then, the first object appeared for 400 ms. Afterward, the empty table was shown for 1 s and the second object appeared for the other 400 ms. Then the virtual desk disappeared, and after a 1 s blank, the word indicating the corresponding question (“you”, “bar”) appeared (see Figure 3). The word “YOU” indicated that participants had to provide an egocentric judgment, that is “What object was closest to you?”, whereas the word “BAR” indicated that participants had to provide an allocentric judgment, that is “What object was closest to the bar?”. Participants answered by clicking a button on the keyboard, that was “C” for cube or cone, “S” for sphere and “P” for pyramid.

The 72 trials were presented in four separate blocks. Each block corresponded to a spatial judgment and a specific pair, that is Egocentric Block with Cube-Pyramid, Egocentric Block with Cone-Sphere, Allocentric Block with Cube-Pyramid, Allocentric Block with Cone-Sphere. Each block included 18 trials. The order and sequence of blocks were counterbalanced across participants.

### 2.5. Data Analysis

Data from the three groups of participants were analyzed using ANOVAs and multiple regression analyses. Specifically, the following ANOVAs were carried out:

1. A 3X2 ANOVA with a between-subject factor “Groups” (aMCI vs. AD vs. NC) and a within-subject factor “Ego-Allo” (i.e., egocentric vs. allocentric judgments) on the mean accuracy at the Ego-Allo Task;
2. Two separate one-way ANOVAs with the between-subject factor “Groups” on scores at FAB and TMT, respectively;
3. A 2X2 two-way ANOVA with the between-subject factor “Groups” and a within-subject factor “Forward-Backward” on scores at CORSI test.

Moreover, separate ANOVAs were performed with sex as an added factor on performance in the Ego-Allo Task and neuropsychological tests. In no case did gender differences appear statistically significant (F < 1). Therefore, issues related to participants’ gender differences will not be discussed further.

For all ANOVAs, the Tukey HSD test was used to analyze post-hoc effects and effect sizes were expressed by *η*^2^*_p_*.

In order to clarify the contribution of frontal, attentional and visuo-spatial memory capacity on the ability to represent egocentric and allocentric spatial information, regression analyses were performed. Specifically:

1. Two stepwise forward multiple regression analyses on egocentric and allocentric mean accuracy separately as the criterion, and scores at FAB, TMT, Corsi Forward and Backward as predictors were carried out on the whole sample regardless of the group of participants;
2. The same regression model as above was carried out on each group of participants separately.

## 3. Results

### 3.1. ANOVAs

**Figure 4:**
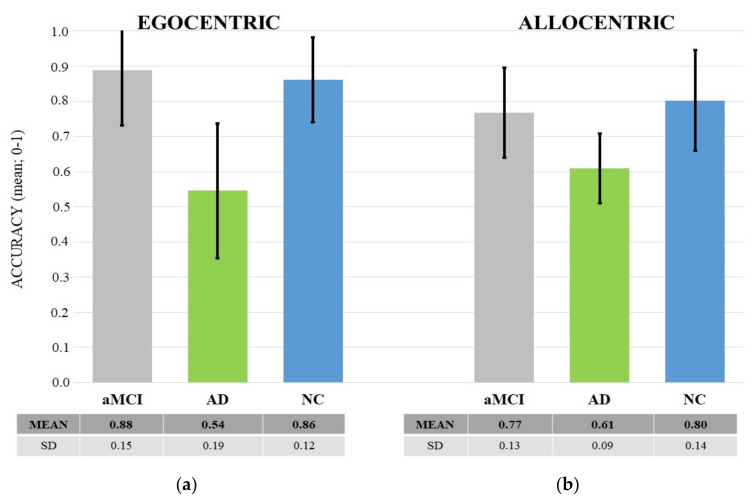
The graph shows the mean accuracy of egocentric (**a**) and allocentric (**b**) judgments as a function of the three groups of participants (aMCI, AD and NC). The black lines represent the standard deviation. Descriptive statistics are also shown in the table below the graph.

Ego-Allo Task. Results showed a main effect of Group: F (2, 49) = 20.13, *p* < 0.0001, *η*^2^*_p_* = 0.45. The post-hoc test showed that ADs performed worse (M = 0.58, SD = 0.15) than both NCs (M = 0.83, SD = 0.15) and aMCIs (M = 0.83, SD = 0.13) (at least *p* < 0.001). An interaction effect between group and Ego-Allo also emerged: F(2, 49) = 4.56, *p* = 0.016, *η*^2^*_p_* = 0.16. The post-hoc test showed that egocentric judgments of ADs were worse than both egocentric and allocentric judgments (at least *p* < 0.01) of aMCIs and NCs. Notably, allocentric judgments of AD patients were less accurate than those of NCs (*p* < 0.005) but did not differ significantly from those of aMCIs (*p* = 0.11). Moreover, aMCIs and NCs showed no significant differences in any of the spatial judgments (*p* > 0.05) (see Figure 4 for descriptive statistics).

### 3.2. Neuropsychological Assessment

**Figure 5:**
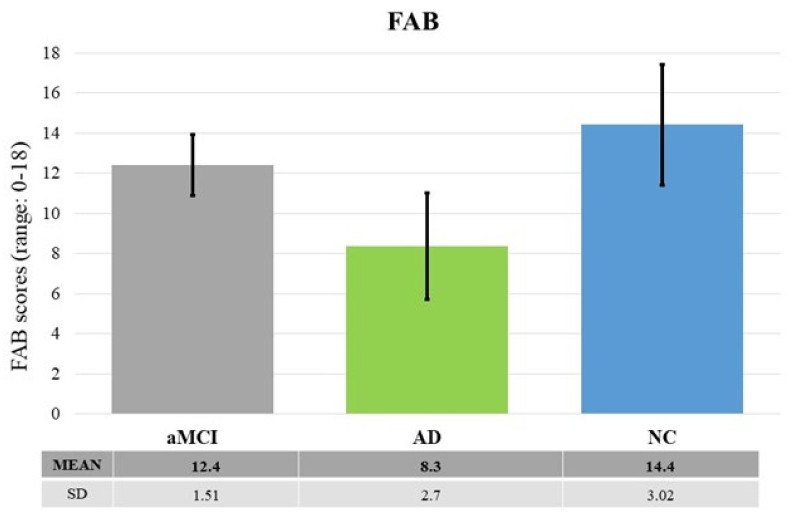
The graph shows the raw scores at FAB as a function of the three groups of participants (aMCI, AD and NC). The black lines represent the standard deviation. Descriptive statistics are also shown in the table below the graph.

FAB. Results revealed significant differences between groups: F (2, 49) = 20.115, *p* < 0.0001, *η*^2^*_p_* = 0.45. The post-hoc test showed that ADs performed worse than both aMCIs and NCs (at least *p* < 0.005). There was no significant difference between NCs and aMCIs (*p* = 0.11) (see Figure 5). 

**Figure 6:**
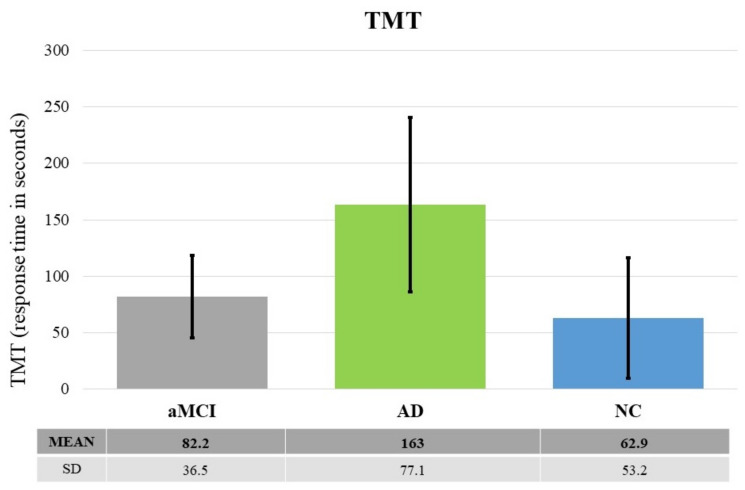
The graph shows the raw scores at TMT as a function of the three groups of participants (aMCI, AD and HC). The black lines represent the standard deviation. Descriptive statistics are also shown in the table below the graph.

TMT. Results revealed significant differences between groups: F (2, 49) = 12.924, *p* < 0.0001, *η*^2^*_p_* = 0.34. The post-hoc test showed that AD patients performed worse than both NCs and aMCIs (at least *p* < 0.01). There was no significant difference between NCs and aMCIs (*p* = 0.62) (see Figure 6).

**Figure 7:**
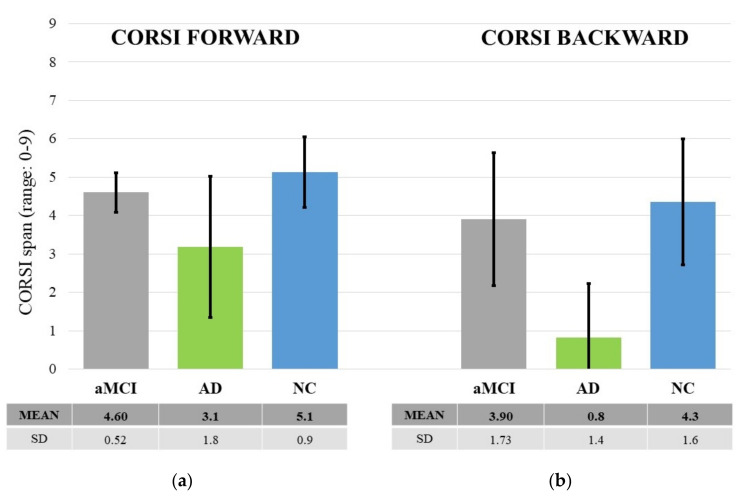
The graph shows the scores at CORSI Forward (**a**) and Backward (**b**) as a function of the three groups of participants (aMCI, AD and NC). The black lines represent the standard deviation. Descriptive statistics are also shown in the table below the graph.

CORSI Block Tapping Test. Results showed a significant main effect of “Groups”: F (2, 49) = 19.71, *p* = 0.000, *η*^2^*_p_* = 0.53. The post-hoc test showed that AD patients performed worse than both NCs and aMCIs (at least *p* < 0.001). There was no significant difference between NCs and aMCIs (*p* > 0.05). There was also a significant “Forward-Backward” main effect (F (1, 49) = 19.712, *p* < 0.000, *η*^2^*_p_* = 0.029): the Corsi span was higher in the forward than backward version. Finally, an interaction between the two factors emerged: F (2, 49) = 3.353, *p* = 0.043, *η*^2^*_p_* = 0.12. The post-hoc test showed that the backward span was shorter in ADs than in NCs and aMCIs (at least *p* < 0.001). Instead, the forward span was shorter in AD patients than NCs (*p* < 0.005), but it did not differ significantly from that of aMCIs (*p* > 0.05). No significant differences appeared between aMCI patients and NCs (*p* > 0.05) (see Figure 7).

### 3.3. Stepwise Forward Multiple Regressions

Egocentric Judgments. The analysis revealed that the overall model was significant: F (2, 49) = 46.15, *p* < 0.0001, R = 0.80, R^2^ = 0.65. As shown in Table 1, the predictors TMT and Corsi Backward contributed significantly to the model: the higher the score at TMT and Corsi Backward, the more accurate the egocentric judgments were.

Allocentric Judgments. The multiple regression analysis revealed that the overall model was significant: F (2, 49) = 21.07, *p* < 0.0001, R = 0.68, R^2^ = 0.46. As shown in Table 2, the predictor FAB contributed significantly to the model: the higher the score at FAB, the more accurate the allocentric judgments were.

aMCI group. The analysis revealed that the overall models for both egocentric and allocentric judgments were not statistically significant (although they approached significance; egocentric: F (3, 6) = 4.71, *p* = 0.051, R = 0.84, R^2^ = 0.70; allocentric: F (2,7) = 3.77 *p* = 0.08, R = 0.72, R^2^ = 0.52). None of the predictors contributed significantly to the models. The final model emerging after the stepwise forward multiple regression procedure is shown in Table 3.

AD group. The multiple regression analysis revealed that the overall models were significant for both egocentric (F (3,7) = 6.02 *p* = 0.02, R = 0.85, R^2^ = 0.72) and allocentric judgments (F (1, 9) = 14.24, *p* = 0.004, R = 0.78, R^2^ = 0.61). As shown in Table 4, the predictor Corsi Backward contributed significantly to the egocentric model: the higher the score at Corsi Backward, the more accurate the egocentric judgments were. Meanwhile, in the allocentric model, the higher the score at FAB, the more accurate the allocentric judgments were. The final model emerging after the stepwise forward multiple regression procedure is shown in Table 4.

NC group. The multiple regression analysis revealed that the overall models were significant for both egocentric (F (4,26) = 4.68 *p* = 0.005, R = 0.65, R^2^ = 0.42) and allocentric judgments (F (1, 29) = 7.38, *p* = 0.01, R = 0.45, R^2^ = 0.20). As shown in Table 5, the predictor TMT contributed significantly to the egocentric model: the better the performance at TMT, the more accurate the egocentric judgments were. In the allocentric model, the higher the score at FAB, the more accurate the allocentric judgments were. The final model emerging after the stepwise forward multiple regression procedure is shown in Table 5.

## 4. Discussion

In the past few years, much evidence has suggested that reduced ability to encode, represent and retrieve visuo-spatial information from memory, especially according to an allocentric reference frame, could be one of the early behavioral markers of the conversion from aMCI to AD dementia [27,38,60,61]. Results of this study add to past evidence. Indeed, patients with AD were overall less accurate than aMCI patients and healthy participants in providing spatial judgments. More specifically, AD patients were less accurate than both aMCI people and healthy participants in providing egocentric judgments. Instead, the last two groups did not significantly differ. This would suggest that the egocentric capacity is well preserved in aMCI. Regarding judgments that required an allocentric reference system, an interesting picture emerged. In fact, AD patients performed worse than healthy people, but not aMCI. In turn, aMCI did not differ even from healthy participants. This finding could further support the idea that an allocentric difficulty could represent the switching point between healthy aging and AD, as anticipated by a condition of aMCI.

However, the novelty of the present work lies in the fact that we sought to clarify the role of attentional-executive and visuo-spatial resources in the egocentric and allocentric encodings. Therefore, we assessed three main executive subcomponents that could be closely linked to the typical functions of these encodings, such as visual attention and planning (TMT), visuo-spatial working memory for retaining and manipulating spatial information (Corsi), and the frontal capacity of inhibiting prepotent responses and monitoring (FAB). In this regard, results showed that AD patients performed worse overall than the aMCI patients and healthy controls at both FAB and TMT. With regard to the Corsi test, it is interesting to note that while in the backward version AD patients performed worse than the other two groups, this did not happen in the forward version. In the latter case, AD patients differed from healthy participants but not from aMCI patients. Therefore, the significant interaction reveals that the backward component, requiring more attentional resources and active processing than its forward version, is seriously impaired in AD patients and constitutes a clear sign of neurodegeneration. Indeed, several studies have revealed that backward span (often called “active” Corsi) is more resource-demanding than forward span (often called “passive” Corsi) [34,62,63] and shows a robust correlation with active spatial tasks such as mental rotation and spatial inference [64]. The ability to remember visuo-spatial information in the forward version also deserves a comment. This ability requires simple retention rather than active processing of information. In this case, the performance of aMCI appears to be somewhere in between that of ADs and healthy people, suggesting the emergence of initial signs of decline.

The results that emerged from the regression analysis may help us to better understand the relationship between participants’ performance on neuropsychological tests and egocentric-allocentric spatial encodings. In general, results showed that attentional resources (TMT) and capacity to actively retrieve visuo-spatial information (Corsi backward) contributed significantly to the egocentric performance. Instead, the allocentric performance was largely linked to frontal executive capacity, with a contribution of the visuo-spatial memory span in its backward version. When examining the single groups, a more specific picture emerged. Although the results should be taken with due caution given the small number of participants within each group (especially aMCI and AD), we believe that they can provide interesting insights. In aMCI patients, the regression models only approached significance. However, they showed that attentional resources, along with the “active” visuo-spatial memory span (Corsi backward) and frontal resources (FAB), explained 70% of the variance in egocentric performance. Moreover, Corsi backward and FAB scores explained 52% of the variance in allocentric performance. In AD patients, “active” visuo-spatial memory span assessed by Corsi backward (mostly), together with attentional resources and “passive” visuo-spatial memory span assessed by Corsi forward, explained 72% of the variance in egocentric performance. Instead, the allocentric performance was substantially and significantly linked to the frontal executive resources, which contributed 61% of the variance. In healthy elderly people, the situation was more varied in relation to egocentric performance. In fact, the four cognitive components assessed here all contributed to the performance, although only attentional resources reached significance. Altogether, the four factors explained 42% of the variance in the egocentric performance. Instead, the allocentric performance was significantly linked to the frontal executive resources that explained the 20% of the variance.

In short, the results confirmed the difficulty of AD patients in egocentric and allocentric judgments. Moreover, the allocentric performance, with aMCI people in the middle between AD patients and healthy elderly people, was confirmed as a crucial spatial symptom of possible AD conversion [21,38]. Interestingly, a similar picture emerged regarding the ’passive’ visuo-spatial memory span (Corsi forward), with aMCI in the middle between AD and healthy controls. Instead, the active Corsi backward, which combines passive spatial memory and active processing requiring attentional resources, gave an important contribution to the egocentric performance. Besides, the frontal resources were largely involved in the allocentric performance. We should consider that the process of normal decline of hippocampal areas causes difficulty in using allocentric representations. This difficulty could lead to a shift from an allocentric to an egocentric strategy [20,38,65] which, in turn, produces a cognitive overload for the frontal areas, which also deteriorate in elderly people and, in particular, AD patients [66,67,68]. The picture is less clear in aMCI, with egocentric and allocentric performances involving visuo-spatial, attentional and frontal resources, but without a significant component strongly and distinctively involved in each spatial task. Probably, aMCIs are still able to strategically use different cognitive resources on which to draw in order to compensate for spatial difficulties, especially allocentric ones.

Recently, Hashimoto and co-workers [69] compared NCs, aMCIs and ADs on a card placing test (CPT) before (part A) and after (part B) a mental rotation task. Results revealed that aMCI patients showed significantly poorer CPT-B performance with respect to the NC group, while AD patients reported significantly poorer performance on both tasks as compared to NC and aMCI patients. The authors concluded that aMCI patients had selective deficits in allocentric encoding, while AD patients were additionally impaired in egocentric encoding. Although Hashimoto and colleagues did not directly measure the ability to use an egocentric or allocentric frame of reference, their results confirmed that allocentric deficits may be a point of contact between the clinical stage preceding the conversion and overt neuropathology. 

It can also be argued that the Ego-Allo task used in the current study does not reflect the complexity of everyday situations faced by people with aMCI and patients with AD. Nevertheless, our results are in line with the results of studies in which more ecological tasks have been used [70,71]. For example, Laczó and colleagues [71] required both aMCI and AD patients to learn a route to reach a specific hidden position within a maze. Participants could use an allocentric strategy to represent distances between the hidden positions and cues in the environment, or an egocentric strategy. These patients, especially ADs, failed to adopt an allocentric strategy (for similar results, see also: [39,72,73,74]). Again, Howett and colleagues [75] observed that patients with prodromal Alzheimer’s disease revealed serious difficulties in a path integration task, i.e., a task that requires updating and keeping track of the egocentric position relative to a reference point. Overall, this evidence is broadly in line with the findings achieved in the current study.

As for the pathological brain changes from aMCI to AD, evidence demonstrates that prior to the appearance of significant clinical symptoms, neurofibrillary changes begin to accumulate in the entorhinal and transentorhinal cortex. Then, an increasing involvement of the medial temporal lobe and surrounding cortical association areas seems to coincide with the appearance of mild clinical symptoms (MCI patients), followed by clinically apparent AD and correspondingly severe worsening of the functioning of all implied areas [76,77]. Therefore, the association of allocentric impairments with early atrophy of the medio-temporal areas (in AD and later in MCI) and the egocentric disorders with impairments in associative and parietal areas in the early stages of the illness (in MCI) can be conceived as early markers of neurodegenerative conversion from aMCI to AD dementia.

In sum, the relationship between frontal resources and allocentric performance could determine the extent to which processes compensate for brain decline in healthy aging, explaining why some older adults age gracefully and others decline rapidly [78]. Regarding AD patients, the decline in frontal resources and the concomitant egocentric difficulty may penalize the functioning of the compensatory processes required for the allocentric damage. Finally, the results suggest that visual-spatial memory, especially when it involves allocentric frames of reference and requires effortful active processing, should be carefully assessed to reveal early signs of possible conversion from aMCI to AD.

## Figures and Tables

**Table 1:** The table shows the relationship between participants’ performance at both TMT and Corsi Backward as predictors and accuracy of egocentric judgments as criteria. The parameters of predictors of the outcome variable are reported.

	Beta	Std. Err	B	Std. Err	t(49)	*p*-Level
TMT	−0.47	0.10	0.00	0.00	−4.83	0.00
CORSI B.	0.46	0.10	0.04	0.01	4.78	0.00

**Table 2:** The table shows the relationship between participants’ performance at both FAB and Corsi Backward and accuracy of allocentric judgments. The parameters of predictors of the outcome variable are reported.

	Beta	Std. Err	B	Std. Err	t(49)	*p*-Level
FAB	0.54	0.13	0.02	0.01	4.29	0.00
CORSI B.	0.21	0.13	0.01	0.01	1.63	0.11

**Table 3:** The upper part of the table shows the relationship between aMCI participants’ performance at FAB, Corsi Backward, TMT (predictors) and accuracy of Egocentric judgments; the lower part of the table shows the relationship between participants’ performance at FAB and Corsi Backward and accuracy of allocentric judgments. The multiple regression parameters are reported.

EGOCENTRIC						
	Beta	Std. Err	B	Std. Err	t(49)	*p*-Level
CORSI B.	0.36	0.27	0.03	0.02	1.35	0.23
TMT	−0.48	0.26	0.00	0.00	−1.84	0.11
FAB	0.34	0.23	0.04	0.02	1.48	0.19
**ALLOCENTRIC**						
CORSI B.	0.47	0.27	0.03	0.02	1.74	0.13
FAB	0.45	0.27	0.04	0.02	1.67	0.14

**Table 4:** The upper part of the table shows the relationship between AD patients’ performance at Corsi Backward, Corsi Forward and TMT (predictors) and accuracy of Egocentric judgments; the lower part of the table shows the relationship between participants’ performance at FAB and accuracy of allocentric judgments. The multiple regression parameters are reported.

EGOCENTRIC						
	Beta	Std. Err	B	Std. Err	t(49)	*p*-Level
CORSI B.	0.78	0.22	0.11	0.03	3.49	0.01
TMT	−0.33	0.21	0.00	0.00	−1.59	0.16
CORSI F.	−0.35	0.22	−0.04	0.02	−1.54	0.17
**ALLOCENTRIC**						
FAB	0.78	0.21	0.03	0.01	3.78	0.00

**Table 5:** The upper part of the table shows the relationship between NC group’s performance at TMT, FAB, Corsi Backward and Forward and accuracy of Egocentric judgments; the lower part of the table shows the relationship between participants’ performance at FAB and accuracy of allocentric judgments. The multiple regression parameters are reported.

EGOCENTRIC						
	Beta	Std. Err	B	Std. Err	t(49)	*p*-Level
TMT	−0.42	0.18	0.00	0.00	−2.39	0.02
FAB	0.16	0.19	0.01	0.01	0.81	0.42
CORSI B.	0.19	0.16	0.01	0.01	1.19	0.25
CORSI F.	0.17	0.16	0.02	0.02	1.09	0.29
**ALLOCENTRIC**						
FAB	0.45	0.17	0.02	0.01	2.72	0.01

## Data Availability

The data presented in this study will be openly available in a publicly accessible repository.
